# Constraints on the modeled vertical distribution of smoke during the 2020 western US wildfires from satellite data

**DOI:** 10.1038/s44407-025-00036-3

**Published:** 2025-12-04

**Authors:** Mackenzie M. Arnold, Pablo E. Saide, Kazuyuki Miyazaki, Kevin W. Bowman, Jordan L. Schnell, Ravan Ahmadov, Xi Chen, Jun Wang, Oscar A. Neyra-Nazarrett

**Affiliations:** 1 Department of Atmospheric and Oceanic Sciences, University of California, Los Angeles, Los Angeles, CA USA; 2 Institute of the Environment and Sustainability, University of California, Los Angeles, Los Angeles, CA USA; 3 Jet Propulsion Laboratory, California Institute of Technology, Pasadena, CA USA; 4 Joint Institute for Regional System Science and Engineering (JIFRESSE), University of California, Los Angeles, Los Angeles, CA USA; 5 Cooperative Institute for Research in Environmental Sciences (CIRES), University of Colorado Boulder, Boulder, CO USA; 6 NOAA Global Systems Laboratory, Boulder, CO USA; 7 Department of Chemical & Biochemical Engineering, The University of Iowa, Iowa City, IA USA

**Keywords:** Climate sciences, Environmental sciences

## Abstract

As wildfires increase in frequency and intensity, accurately representing the vertical distribution of smoke in numerical models is critical for assessing impacts to air quality, but remains highly uncertain. In this study, we leverage satellite retrievals of total column carbon monoxide (CO) and aerosol layer height (ALH) to evaluate two state-of-the-art regionals and global models, one using a plume rise parameterization to estimate smoke injection height (RAP-Chem) and another placing smoke at the surface (MOMO-Chem). We introduce a novel metric that utilizes the differing vertical sensitivities of two satellite sensors observing CO (TROPOMI and CrIS) to infer the vertical distribution of wildfire smoke using a joint CO column ratio. We find that RAP-Chem better captures the distribution of CO and ALH related to the 2020 western US megafire event than MOMO-Chem. However, RAP-Chem underestimates surface CO concentrations, revealing that current plume rise parameterizations are limited in their ability to partition smoke correctly in the vertical column. These results show that synergistic use of satellite data can provide additional constraints on the vertical distribution of smoke, thus providing insights into the strengths and limitations of current plume rise parameterizations and a pathway to improvement.

## Introduction

Wildfires are becoming more frequent and extreme^1,2^ due to climate change and weather extremes^3^, poor forest management, expansion of the wildland-urban interface, and increased anthropogenic ignitions^4^. This trend is projected to continue globally^5,6^ and the effects will extend far beyond the region burned. As wildfires grow larger and more intense, they create more extensive smoke plumes that rise higher in the atmosphere^7^, and affect larger areas and populations. Here, we will consider “smoke” in a broad sense to include all wildfire emissions including aerosols and trace gasses^8^. Climate effects of wildfire smoke include atmospheric temperature anomalies^9^, increased loads of black carbon aerosols and trace gases^10^, altered radiative budgets^11^, altered albedo^12^, and implications for hydrologic cycles^13^. Smoke that reaches the lower stratosphere via convective pyrocumulonimbus clouds can induce climate effects similar to that of a moderate volcanic eruption^14–16^. Wildfire smoke also poses a health hazard and has been linked to increased respiratory and cardiovascular disease^8,17^. During the summer of 2020, a series of large wildfires burned over 10.2 million acres in the Western United States, resulting in 3720 exceedances of the National Ambient Air Quality Standards for fine particulate matter (PM_2.5_)^18^. The resulting smoke plumes were transported across the United States and reached Europe by mid September^19^.

Wildfire plume rise, caused by combustion heat-flux driven buoyancy, influences the altitude where aerosols and gasses are emitted by wildfires and injected into the atmosphere. The plume injection height plays a major role in determining the transport pathways and extent of smoke plumes. Injections within the planetary boundary layer (PBL) will result in more local air quality effects^20^. In contrast, injections above the PBL can result in regional or even continental transport. Subsequent boundary layer entrainment can significantly affect near surface air quality hundreds of kilometers away^21,22^. Plume injection height is driven by a complex combination of fire size, fire heat flux, and atmospheric conditions^23^. Intense fires tend to result in higher plume heights^20,24^. However, ambient meteorological conditions, including atmospheric stability^20^, horizontal winds^25^, and moist convection^20^, also strongly influence the plume dynamics and eventual plume height.

It is critically important to be able to accurately simulate and predict smoke transport in order to understand and mitigate the effects. However, wildfire plume rise remains difficult to represent in atmospheric models due to persistent uncertainties and complex physical interactions taking place on small scales that can’t be explicitly resolved in regional and global models due to large vertical and horizontal grid spacing. An analysis of twelve state-of-the-art models showed substantial discrepancies in simulated plume heights (up to 5 km) for the same fire event^26^. Consequently, air quality forecasts vary between different models and parameterizations. For example, three widely used plume rise parameterizations have shown up to 20–30% difference in near-source aerosol optical depth (AOD) and PM_2.5_ concentrations and 5% difference downwind^27^.

Verification and improvements to plume rise parameterizations are made difficult due in part to the limited spatial and temporal scale of observations of smoke vertical distribution. Observations of wildfire smoke have been obtained through a range of platforms, including in situ and remote sensing surface sensors, aircraft campaigns, and satellite instruments. AOD or surface particulate matter (PM) measurements are commonly used to verify wildfire smoke concentrations, however, these measurements alone don’t provide information on the vertical distribution of smoke and are not sufficient to verify plume height. In addition to aerosols, carbon monoxide (CO) is frequently employed as a tracer for wildfire smoke^28–31^ due to its association with incomplete combustion^31^, long lifetime in the atmosphere^32^, and insolubility^33^. CO and biomass burning aerosols are correlated but CO also offers several advantages over AOD as its concentrations do not saturate at high concentrations^34^. Furthermore, AOD is an optical property and the assumptions that need to be made to convert to mass concentration can change substantially with smoke age^35^, and cannot currently be reproduced in models. The most detailed information about the vertical profile can be retrieved using Lidar, but these instruments have various limitations. Spaceborne Lidars have a very fine vertical resolution but a very narrow ground track which greatly limits the number of fires with data available^27,36,37^. Furthermore, some of these lidars are no longer operational^36,38^. Airborne Lidar measurements have been used to evaluate smoke plume height but are even more limited in temporal and spatial scale^26,39,40^. Satellite-derived aerosol layer height (ALH) retrievals have a higher spatial coverage than Lidar, but a more limited vertical resolution as they only retrieve the top or centroid height of an aerosol plume. ALH retrievals measured using stereoscopic techniques from instruments such as the Multi-angle Imaging SpectroRadiometer (MISR)^41^, have been used to evaluate modeled plume heights^20,27,42–44^. However, peak fire activity tends to occur in the late afternoon^45,46^, so MISR, with a morning overpass time, tends to have a bias to low altitude plumes^20^. The TROPOspheric Monitoring Instrument (TROPOMI)^47^, which utilizes spectroscopic techniques based on oxygen bands to retrieve the aerosol optical central height (AOCH), has a daily early afternoon overpass time and shows promise to verify plume heights. Retrievals of CO total column and CO vertical profiles using optimal estimation from the TRopospheric Ozone and its Precursors from Earth System Sounding (TROPESS) Cross-track Infrared Sounder (CrIS) are another valuable resource to evaluate smoke vertical distribution.

This study uses remote sensing retrievals of CO and ALH to verify the modeled spatial and vertical distribution of wildfire smoke during the 2020 Western U.S. wildfires. We use CO total column retrievals from TROPOMI and CrIS to evaluate smoke transport and introduce a novel joint satellite ratio to extract additional information about the vertical distribution of smoke. We also use high-resolution TROPOMI AOCH retrievals, which provide the central height of the aerosol layer, to evaluate plume height. We use these datasets to evaluate the performance of two modeling systems: (1) NOAA’s experimental Rapid Refresh with Chemistry (RAP-Chem) model, which incorporates the Freitas plume rise parameterization, and (2) NASA’s Multi-mOdel Multi-cOnstituent Chemical data assimilation (MOMO-Chem) system, which does not include a plume rise parameterization. We present model-observation comparisons for CO total column, surface CO, ALH, and the joint CrIS/TROPOMI ratio. In particular, we explore the synergistic effects of analysis using ALH and the CrIS/TROPOMI ratio to understand the vertical distribution of smoke. Finally, we discuss pathways to improve model depiction of the vertical distribution of smoke.

## Results

### Comparison of CO total column

**Fig. 1:**
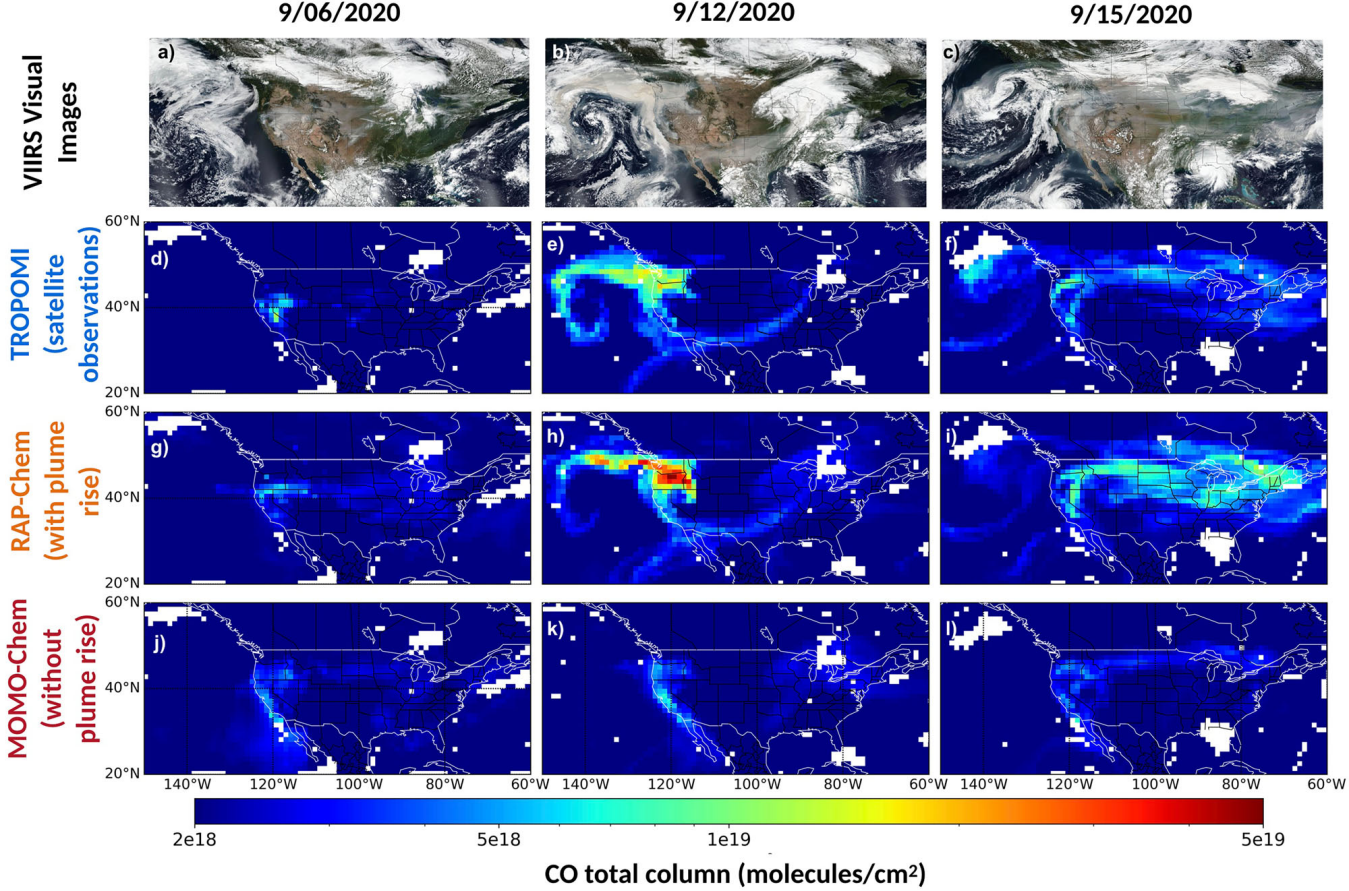
Maps of visible imagery and the modeled and observed CO total column. **a-c** Visible Infrared Imaging Radiometer Suite (VIIRS) Visual Images, (**d-f**) TROPOMI CO total column, (**g-i**) RAP-Chem CO total column with TROPOMI averaging kernel applied, and (**j-l**) MOMO-Chem CO total column with TROPOMI averaging kernel applied on September 6, 12, and 15, 2020. Units are molecules/cm^2^. The VIIRS Visual Images are NASA Worldview snapshots (https://wvs.earthdata.nasa.gov).

First, we compared the CO total column retrieved from TROPOMI against the model derived CO total column. Figure 1 shows the visual smoke from Visible Infrared Imaging Radiometer Suite (VIIRS) retrievals and spatial plots of the CO total column for TROPOMI and both models for a relatively low fire activity day (September 6th, 2020), a high fire activity day with transport over the Pacific Ocean (September 12th, 2020), and a strong eastward transport day (September 15th, 2020). It is visually evident from Fig. 1 that RAP-Chem captures the observed high CO plumes in all cases better than MOMO-Chem. RAP-Chem captures the observed spatial patterns, depicting both the westward plume over the Pacific Ocean and the subsequent eastward transport across the country, with the spatial correlation R^2^ of 0.26 with TROPOMI and a R^2^ of 0.19 with CrIS (spatial plots shown in Supplementary Fig. S1). This behavior indicates the strength of this plume rise parameterization in placing the plume in the vertical column accurately enough to capture the general pattern of transport, but the weak correlations indicate remaining biases likely associated with smoke emission uncertainties, and errors in the vertical distribution and transport of smoke. While RAP-Chem captures the spatial distribution well, it does tend to overestimate the CO total column in some regions by nearly a factor of 2, particularly near regions of high intensity fire activity such as over Oregon on September 12th. It should be noted that because RAP-Chem is a forecast model, the fire emissions are based on the satellite observations of fires for the previous day and therefore we would expect inconsistencies in the emissions.

In contrast, MOMO-Chem performs with lower skill, particularly in the high fire activity days. In every day analyzed with significant fire activity and/or smoke transport, MOMO-Chem underestimated both the spatial extent of CO and the emissions due to wildfire activity, resulting in R^2^ values below 0.07 between the TROPOMI CO total column and the MOMO-Chem CO total column. The underestimation of up to 2 × 10^19^ molecules/cm^2^ occurs during periods of high fire activity. This behavior is likely a combination of underestimated emissions and/or observational constraints, the coarser resolution in their global analysis, and the lack of plume rise parameterization. This analysis shows that even a high-quality global reanalysis model underperforms in extreme fire events because it was not designed to resolve these events. RAP-Chem’s much better performance highlights the importance of including plume rise parameterizations when modeling wildfire emissions in order to accurately depict the horizontal transport.

### Comparison of vertical profile metrics

**Fig. 2:**
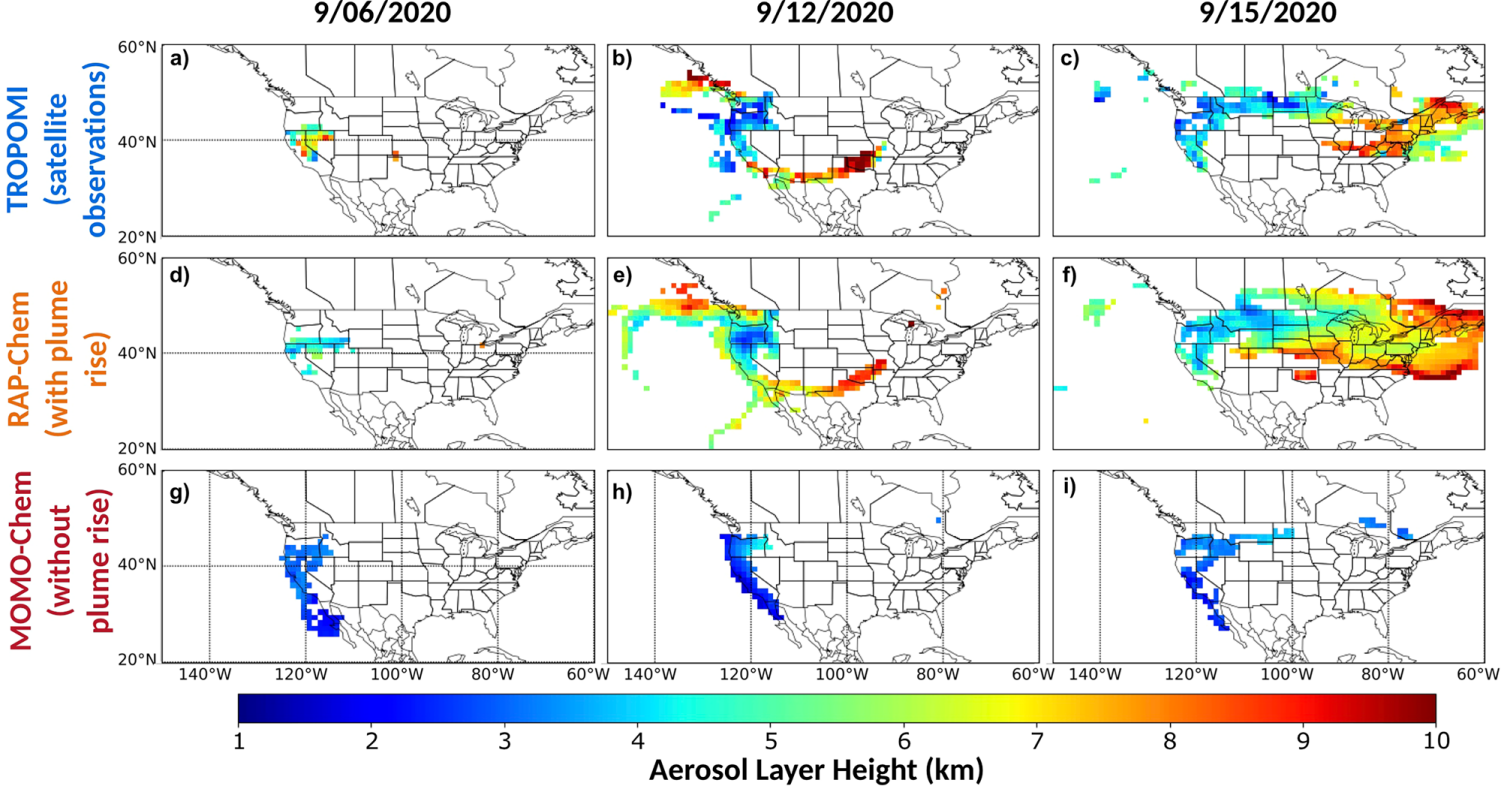
Maps of the modeled and observed aerosol layer height. **a-c** TROPOMI aerosol optical central height, (**d-f**) RAP-Chem aerosol layer height, and (**g-i**) MOMO-Chem aerosol layer height on September 6, 12, and 15, 2020. Data is masked by smoke using a CO total column threshold of 3 × 10^18^ molecules/cm^2^.

**Fig. 3:**
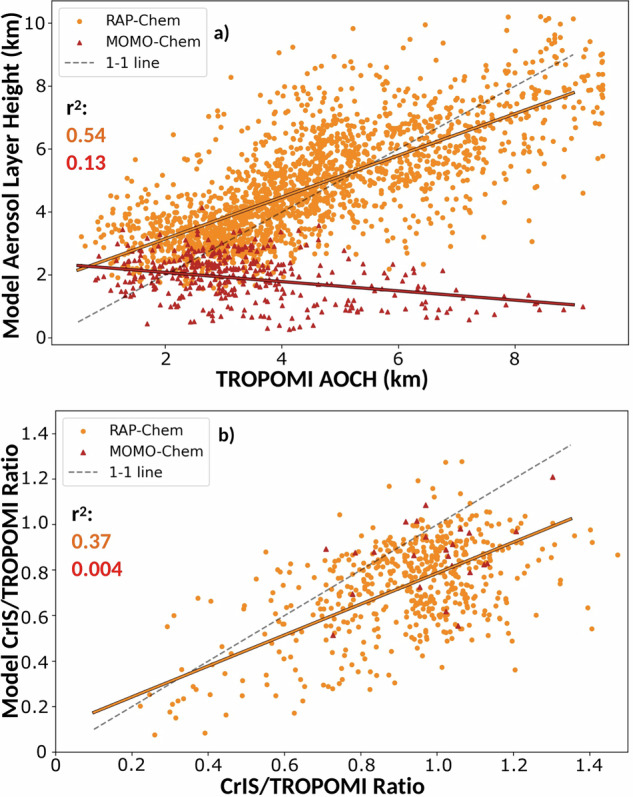
Comparison of the modeled and observed ALH and CrIS/TROPOMI ratio. **a** Correlation between the TROPOMI aerosol optical central height (km) and the model aerosol layer height. **b** Correlation between the CrIS/TROPOMI ratio and the model equivalent CrIS/TROPOMI ratio. RAP-Chem and MOMO-Chem are shown in orange and red respectively. Data is masked by smoke using a CO total column threshold of 3 × 10^18^ molecules/cm^2^. The solid color lines show the linear least squares correlation between the observed and modeled variable.

Figure 2 shows the spatial plots of ALH on Sept. 6th, 12th, and 15th. The TROPOMI AOCH shows lower heights (2–4.5 km) in the regions closest to the fires and higher plume heights (7–9 km) in cases of long-range transport away from the fires. The RAP-Chem ALH shows similar general trends as the TROPOMI AOCH and appears to be reproducing elevated and lower plumes. However, RAP-Chem also appears to underestimate the height of extremely elevated plumes (8–9 km), such as over the Pacific Ocean just off the coast of Canada on Sept. 12th, and overestimate shallower plumes (1–3 km), such as over Oregon and Washington on Sept.12th. This can be seen more clearly in Fig. 3a. This agrees with previous studies with models using the Freitas plume rise scheme^24,26,39^. Figure 3a also shows a clear difference between the RAP-Chem and MOMO-Chem aerosol layer height. While the RAP-Chem ALH is correlated with TROPOMI AOCH with an R^2^ value of 0.54, MOMO-Chem shows low correlation (R^2^ of 0.14) with the observational dataset with all MOMO-Chem values being below 4 km. This result is expected given the highly convective nature of this wildfire event and MOMO-Chem not including plume rise, and thus, explains much of the differences in CO transport^42^. RAP-Chem ALH has a mean bias error of 0.32 km compared with the observations which is within the reported uncertainty range of TROPOMI AOCH (0.5 km). RAP-Chem’s plume rise parameterization allows it to better capture the aerosol layer height over the time period observed. While this metric is useful to determine the approximate height of an aerosol layer, it does not provide any information on the vertical distribution of the plume.

**Fig. 4:**
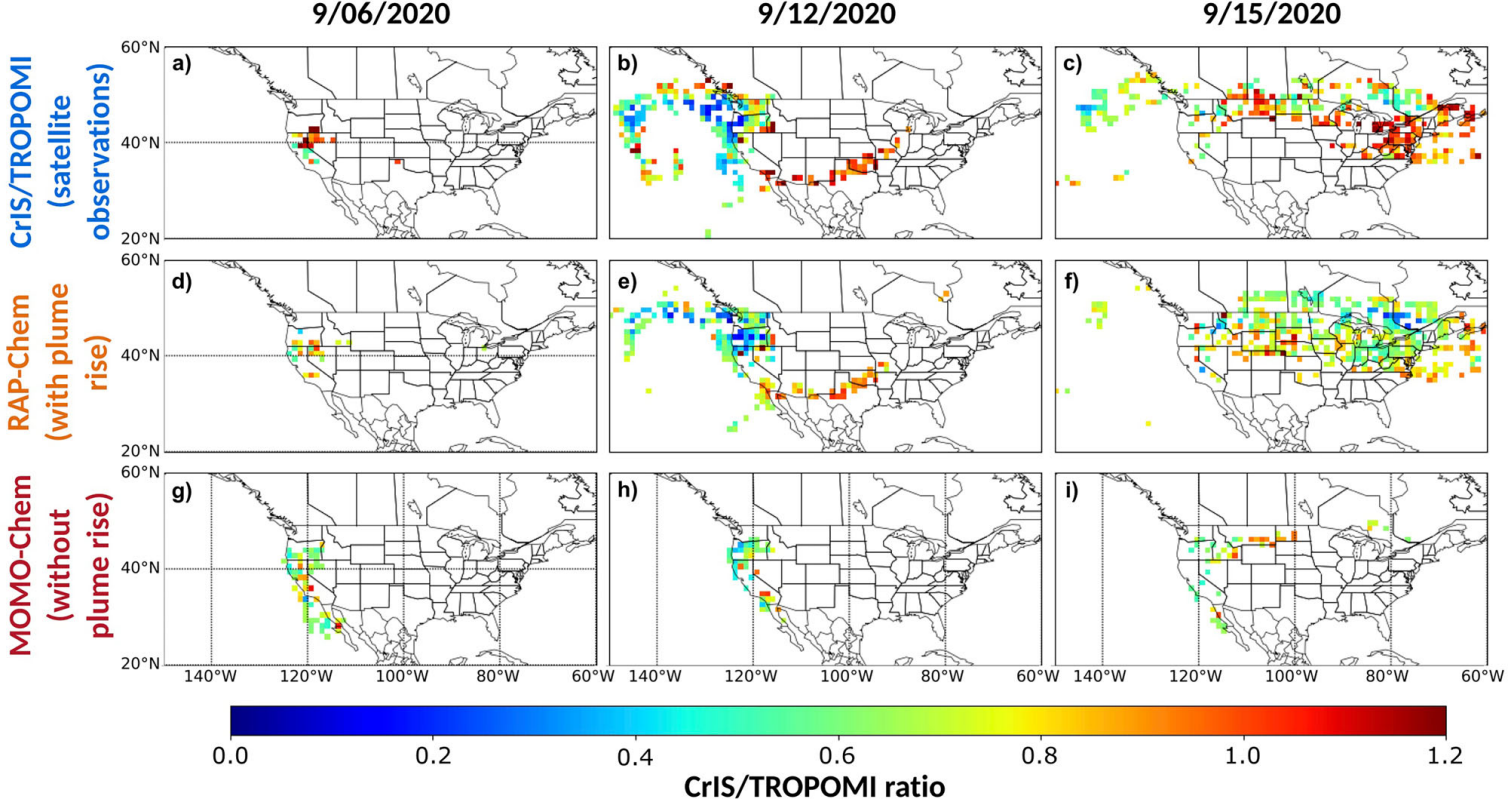
Maps of the modeled and observed CrIS/TROPOMI ratio. **a-c** CrIS/TROPOMI ratio, (**d-f**) RAP-Chem CrIS/TROPOMI ratio, and (**g-i**) MOMO-Chem CrIS/TROPOMI ratio on September 6, 12, and 15, 2020. Data is masked by smoke using a CO total column threshold of 3 × 10^18^ molecules/cm^2^.

The upper panels in Fig. 4 show spatial plots of the CrIS/TROPOMI ratio on Sept 6th, 12th, and 15th. The ratio describes the percentage of the CO total column at the altitudes where the CrIS vertical sensitivity (calculated using Eq. (6)) peaks, which can give information about the altitude of smoke. There tends to be lower ratios in regions with lower aerosol layer height. This is what we would expect as the CrIS averaging kernel tends to be more sensitive at altitudes between 6 and 9 km (Supplementary Fig. S2). The models tend to underestimate the ratio (Fig. 3b) implying inconsistencies with the placement and distribution of the plume. RAP-Chem CrIS/TROPOMI ratio has a R^2^ value of 0.37 and shows a better correlation with the observations than the MOMO-Chem ratio does. This is lower than the correlation for the RAP-Chem ALH but higher than the R^2^ correlation for the CO total column comparison with TROPOMI. Some of this difference could be due to errors in distribution despite accurate plume placement and some due to downwind effects of inaccurate plume placement. Despite releasing all biomass burning emissions at the surface, MOMO-Chem has a higher ratio than expected in many cases. This is because MOMO-Chem surface CO enhancements due to smoke are low compared to the free-tropospheric background concentration; and thus, there isn’t a large difference between the total column when the TROPOMI or CrIS averaging kernel is applied, resulting in a relatively high ratio.

**Fig. 5:**
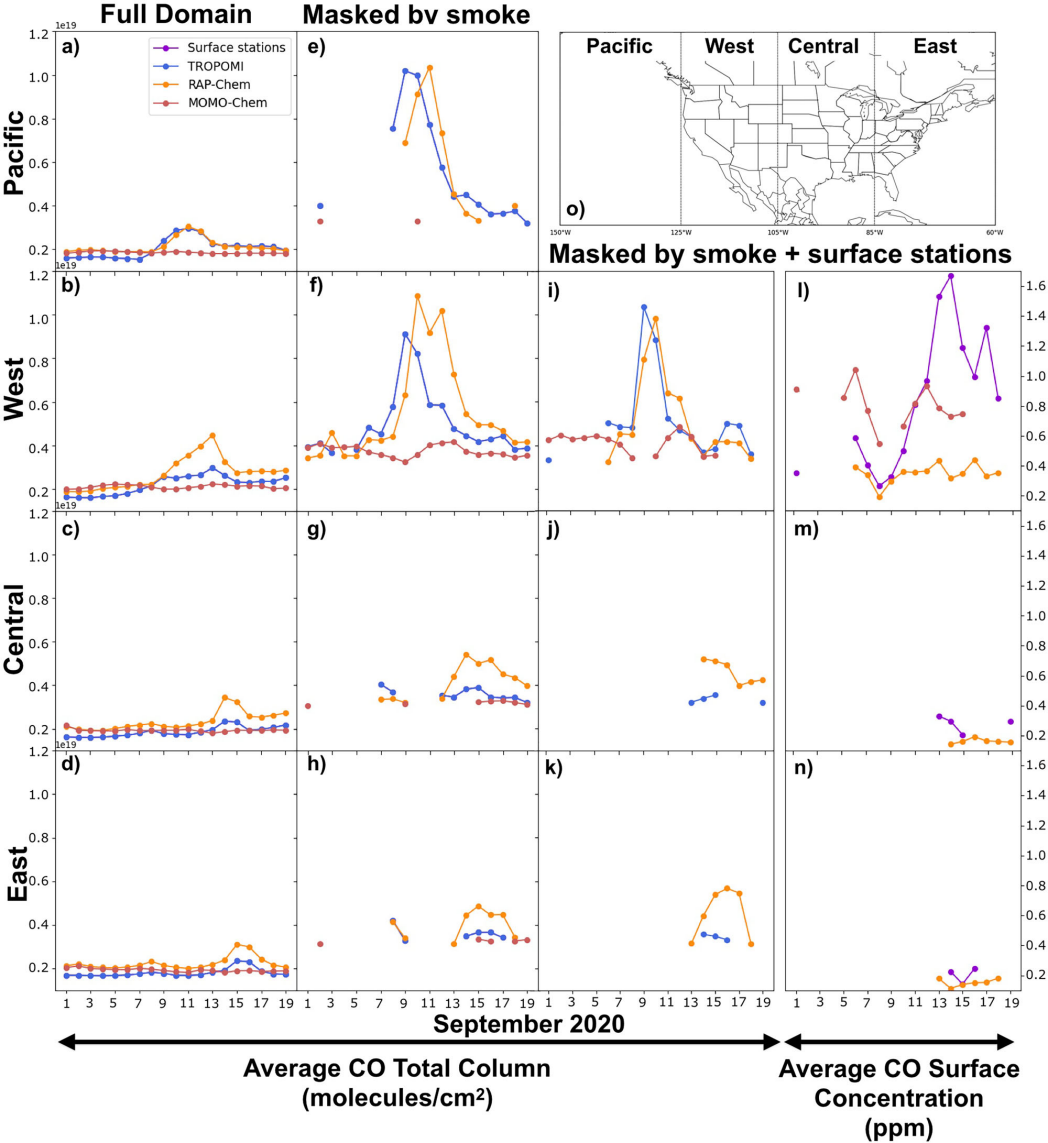
Daily average timeseries of modeled and observed CO total column and surface concentration grouped by region. Daily average CO total column in molecules/cm^2^ for TROPOMI, RAP-Chem and MOMO-Chem averaged over the full regional domain (**a**–**d**), masked by smoke (**e**–**h**), and masked by smoke and surface CO stations (**i**–**k**). Daily average CO Surface Concentration for surface CO stations, RAP-Chem and MOMO-Chem (**l**–**n**). Model CO total columns have the TROPOMI averaging kernel applied. Smoke mask is defined by a CO total column threshold of 3 × 10^18^ molecules/cm^2^. Regions are defined as followed and shown in (**o**)): Pacific (150W–125W), West (125W–105W), Central (105W–85W), East (85W–60W).

Time series of the average CO total column and surface CO concentrations for models and observations in different regions is shown in Fig. 5. Various masks are applied in the different columns. “Full domain” takes the average over the entire regions, both smoky and non-smoky areas. The “smoke” mask was determined by setting a CO threshold of 3 × 10^18^ molecules/cm^2^. This is above regular background CO levels, so the average only includes regions strongly affected by wildfire smoke. The “surface stations” mask takes the average over model points collocated to the location of surface CO stations to allow for direct comparison with the observations. The regions are defined according to Fig. 5o. When averaged over the full domain (Fig. 5a–d), RAP-Chem accurately captures the CO total column in the Pacific region and tends to overestimate the total column in the West, Central and East. The peak of the total column is on September 12th in the Pacific region and shifts a day later in each subsequent region as the smoke is transported eastward. When averaged over smoky regions (Fig. 5e–h), RAP-Chem tends to underestimate the initial peak in CO and overestimate the later peak in the Pacific and West regions. This lag may be related to RAP-Chem being a forecast model. There is only one RAP-Chem initialization per day, and the emissions are based on the persistence method using FRP from the previous 24 h. Although the observations are more sparse, RAP-Chem shows similar performance for the CO total column when co-located to surface stations (Fig. 5i–k). However, despite this overestimation of the total column, RAP-Chem tends to underestimate the surface concentration of CO (Fig. 5l–n), which is more pronounced after Sept. 9 when the CO columns are overpredicted. In contrast, MOMO-Chem underestimates the CO total column over all regions and masks but when compared against the observed surface CO, MOMO-Chem has a lower mean bias error (−0.19) than RAP-Chem (−0.36). MOMO-Chem does not have enough CO in the total column but because all the emissions are placed right at the surface, the model better realizes surface concentrations. Similar results were found for time series comparisons with CrIS (Supplementary Fig. S3).

Additionally, in the West region when masked by “smoke+surface stations”, the observed surface CO peaks when the observed CO column is at a minimum on Sept. 13–15. As the surface stations tend to be located in urban areas that are primarily affected by transported smoke, this implies that during periods of peak fire activity when the CO column is the highest, the transported smoke is lofted and has less of an effect on surface air quality a distance from the fire. The elevated surface CO from Sept. 13–15 exceed 1.6 ppm and coincides with observed CO columns that are within the lowest during this period with minimum values of 3.7 × 10^18^ molecules/cm^2^, indicating that periods of lower biomass burning emissions could result in weaker smoke plumes that remain confined to the boundary layer and have a greater impact on regional surface air quality. As stated earlier, RAP-Chem is likely overpredicting the injection height during this period and injecting the smoke above the boundary layer where it has less of an effect on surface air quality.

**Fig. 6:**
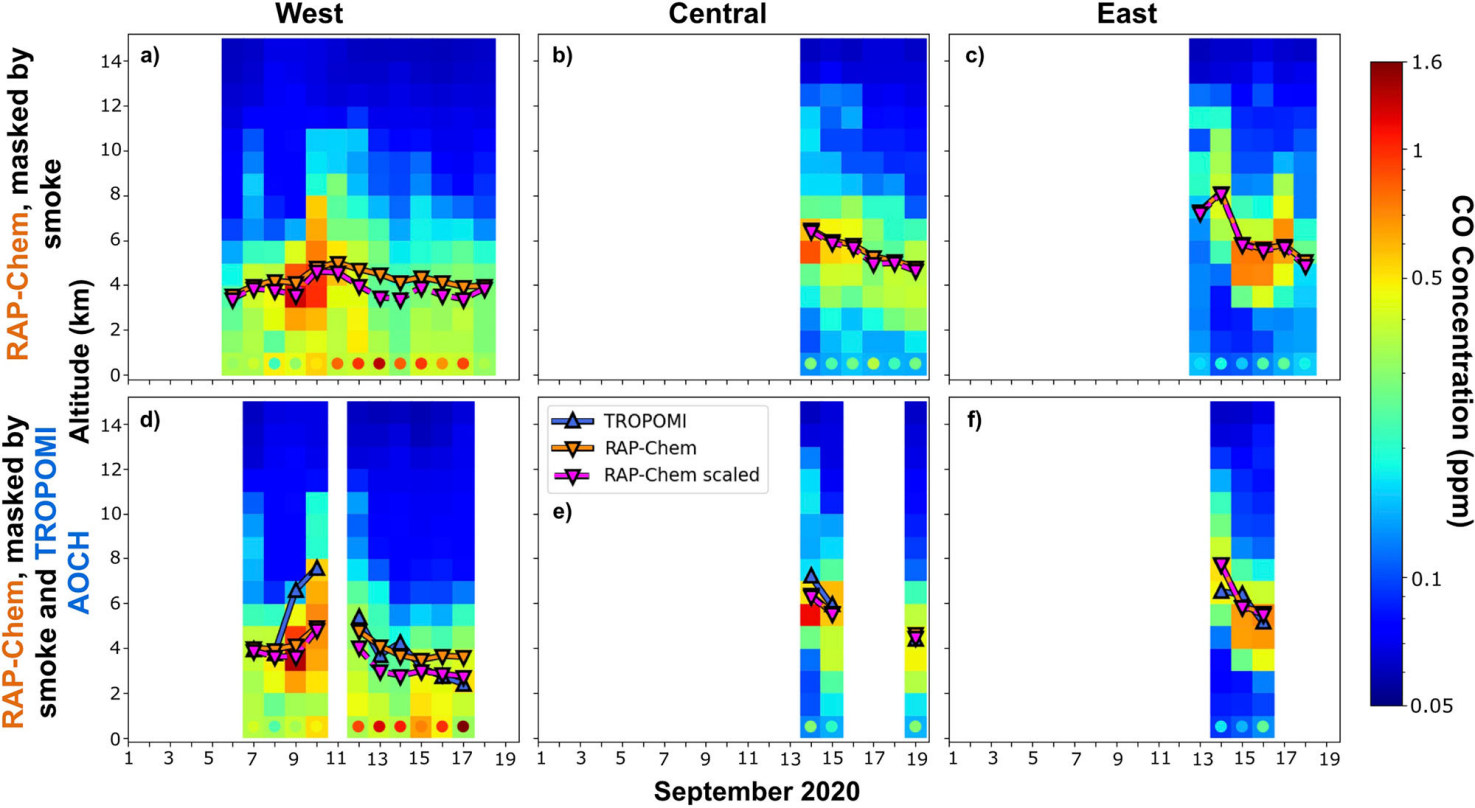
Daily average modeled vertical profiles and comparison of modeled and observed ALH. Daily average RAP-Chem CO vertical profiles in units of ppm averaged over regional domains. The circles in the lowest level depict the daily average CO concentrations retrieved from observational surface stations. The daily average RAP-Chem ALH and TROPOMI AOCH are overlayed on top of the vertical profiles. The scaled RAP-Chem line is the ALH calculated after scaling the RAP-Chem surface CO concentration to the observational surface CO concentrations. All data is masked by smoke defined as above a CO total column threshold of 3e18 molecules/cm^2^ and colocated to the location of the surface CO stations **(a-f)**. The lower row is also masked by TROPOMI AOCH to account for the gaps in data **(d-f)**.

Additional analysis was performed to determine if RAP-Chem’s inconsistency in capturing the surface CO concentration is due to incorrect vertical placement of the plume. We only considered RAP-Chem in this analysis due to MOMO-Chem’s underestimated CO total column and ALH. Figure 6 shows a time series of RAP-Chem’s average vertical profile of CO over the smoky region overlaid with the original aerosol layer height. Additionally, a recalculated aerosol layer height is displayed, calculated after scaling the surface concentrations of RAP-Chem to the observations. As RAP-Chem tends to underpredict surface concentrations, the scaled ALH tends to be lower than the original ALH. The R^2^ value between TROPOMI AOCH and RAP-Chem ALH increases from 0.38 to 0.39 when the model surface concentrations are scaled to observations but the mean bias error increases in magnitude from −0.43 to −0.82. The correlation shows very little improvement and the bias in the model compared to the observations increases which suggests that the model biases in surface concentration are unrelated to biases in the model ALH. Another way to interpret these results is that assimilation of ALH would likely not result in significant improvement of surface concentration for this type of event.

RAP-Chem demonstrates the limitations of current plume rise parameterizations and the reasons many models don’t include them. A model can get close to the total column amount of CO and place the plume at approximately the correct height but still get the surface concentrations incorrect. This implies that the RAP-Chem’s inconsistent surface CO concentrations are less dependent on the injection height of the plume rise parameterization and more dependent on other factors, such as the partition of emissions between the injection height and the surface^40^. While the partition used by RAP-Chem is a fixed number (90% spread between the lower and upper bounds of injection height) that is similar to those constrained with airborne observations^40^, it’s likely that the partition varies with conditions and future work should explore developing parameterizations for this partition.

### Synergy between ALH and CrIS/TROPOMI Ratio

**Fig. 7:**
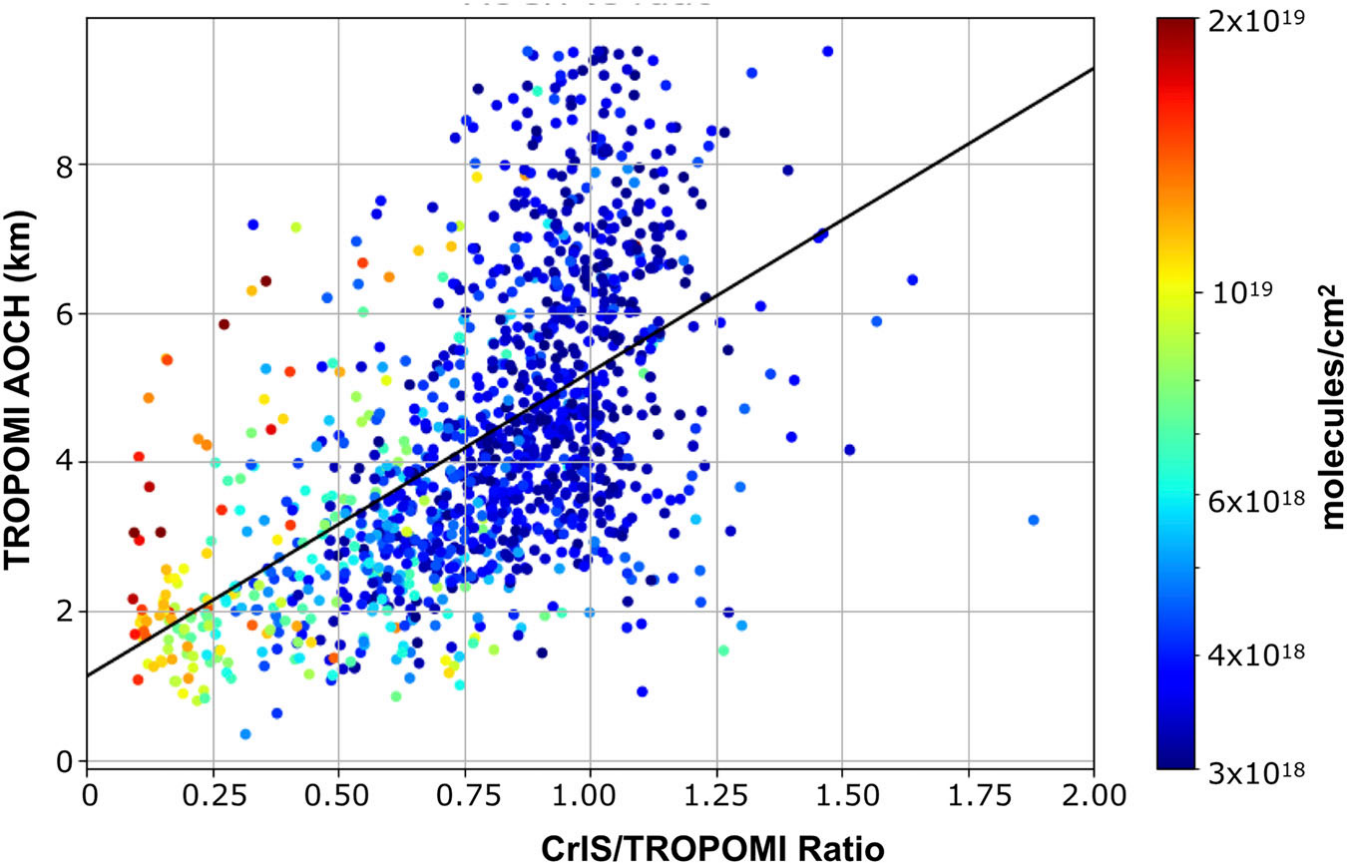
Comparison of the CrIS/TROPOMI ratio and TROPOMI AOCH. Correlation between the CrIS/TROPOMI ratio and the TROPOMI AOCH (km). The black line shows the linear least-squares fit. The colors show the TROPOMI CO total column in units of molecules/cm^2^. Data is masked by smoke using a CO total column threshold of 3 × 10^18^ molecules/cm^2^.

Aerosol layer height and the CrIS/TROPOMI ratio are useful metrics for verifying smoke plume height individually, but when analyzed together they have the potential to provide additional information about the vertical profile. ALH retrieves a single height that shows where in the vertical profile the plume is, however, it doesn’t give a detailed vertical profile. With this metric alone, we have no way of knowing if the plume is concentrated in a shallow layer or spread vertically over several km. Additionally as ALH acts similar to a weighted average, it performs poorly in cases where there are multiple plumes at different altitudes^48^. The CrIS/TROPOMI ratio retrieves the fraction of the column within a certain altitude range which provides information about plume distribution. Depending on the location of the plume and CrIS sensitivity, the usefulness of this ratio varies on a case-by-case basis. These metrics are measurements of two different quantities (CO and aerosols), but as they are co-emitted from wildfires, they are related (R^2^ value of 0.31, Fig. 7). Low total columns tend to have consistently high ratios due to increased influence of the background concentration (Fig. 7). This will affect the CrIS/TROPOMI ratio more than the ALH because CO has a higher relative background concentration than aerosols. Combining the ALH and the ratio provides information on both the height and distribution of the plume and can be a powerful verification tool.

**Fig. 8:**
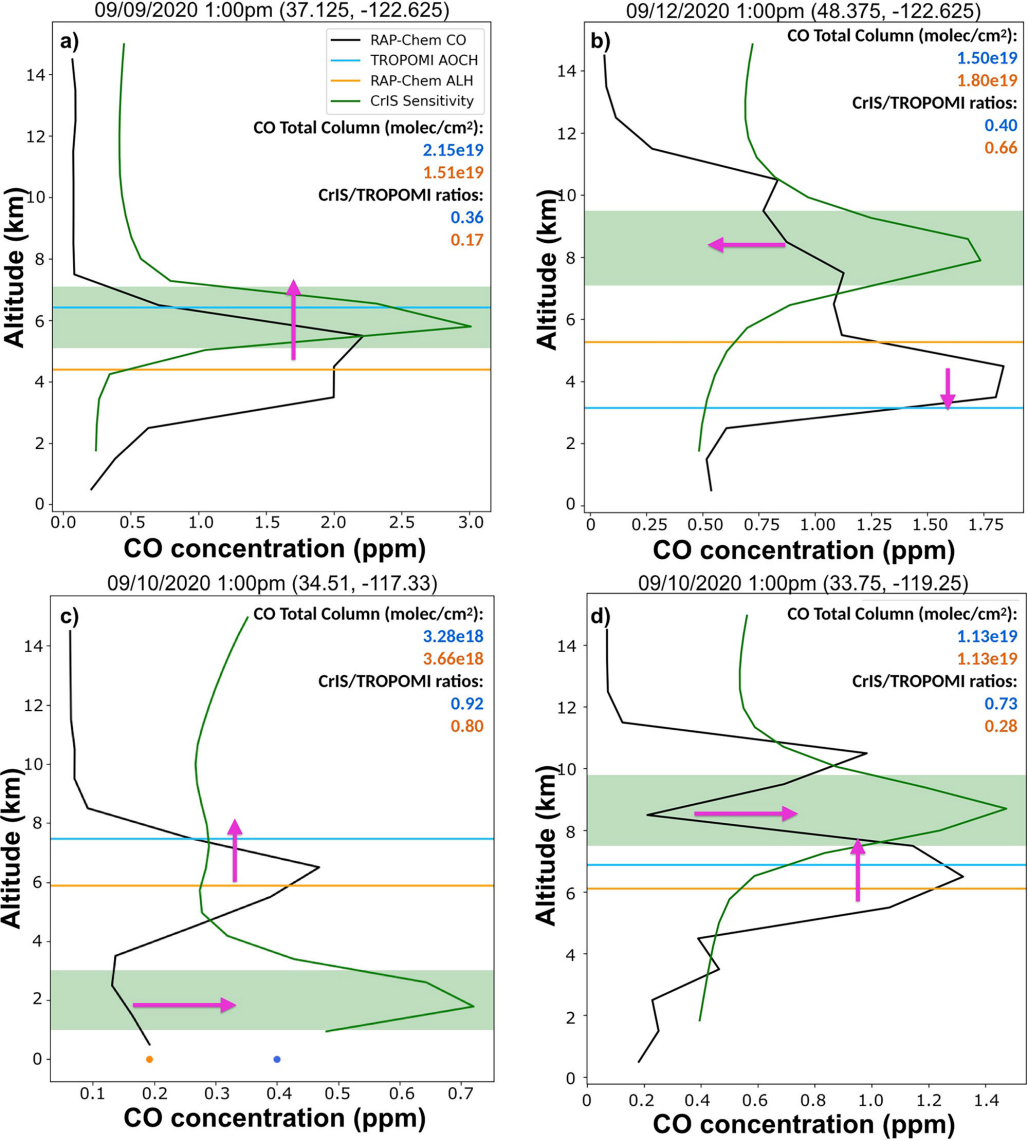
Verification of modeled vertical profiles using aerosol layer height and the CrIS/TROPOMI ratio. Vertical profiles of the RAP-Chem CO (black), TROPOMI AOCH (blue), RAP-Chem ALH (orange) and relative CrIS sensitivity (green) for four different cases **(a-d)**. Green shaded region depicts the altitudes of CrIS highest sensitivity calculated using Eq. (5). Pink arrows depict how the RAP-Chem vertical profile could be changed to increase consistency with TROPOMI AOCH and the CrIS/TROPOMI ratio simultaneously. The orange and blue circles at ground level are the RAP-Chem and the observed surface CO concentration respectively. The orange and blue numbers show the CO total column and the CrIS/TROPOMI ratios for RAP-Chem and TROPOMI respectively.

The synergy between ALH and the CrIS/TROPOMI ratio can be seen in a case study of vertical profiles. Figure 8a shows an elevated plume where RAP-Chem is underpredicting both the ALH (by ~2 km) and the CrIS/TROPOMI ratio (0.17 modeled vs. 0.36 observed). The lower model ALH suggests that the model is putting the plume too low and that the bulk of the plume needs to be moved to a higher altitude to match the observations. In addition, the lower CrIS/TROPOMI model ratio implies that CO needs to be increased at the altitudes where CrIS is most sensitive (5–7 km for this case, see definition in Methods). This profile is an example of the simplest case where one adjustment to the plume (move it higher) would improve consistency with both the ALH and the CrIS/TROPOMI ratio simultaneously. However, many profiles are not this simple. Figure 8b shows an elevated plume where the model is overpredicting the ALH and CrIS/TROPOMI ratio. The difference in ALH implies that the observed plume is lower than the modeled plume. However, as RAP-Chem has a higher ratio than the observations, this implies that there is too much CO in the area CrIS is more sensitive (6–10 km). Simply shifting the model plume lower to match the ALH would not eliminate the bias as the ratio suggests that the concentration of CO at higher altitudes also needs to be lowered. For this profile, two separate actions (see two arrows in Fig. 8b) related to both plume height and relative concentrations need to be taken to improve the consistency with TROPOMI AOCH and CrIS/TROPOMI ratio.

Figure 8c shows a seemingly simple modeled plume structure that peaks at an altitude of 6.5 km with the model underpredicting both the ALH and the CrIS/TROPOMI ratio. The modeled plume would need to be moved upwards to improve consistency with TROPOMI AOCH, however, to improve consistency with the CrIS/TROPOMI ratio, more of the modeled plume would need to be moved downward to the altitudes that CrIS is most sensitive (1–3 km). To reconcile these seemingly contradicting changes and improve both the ALH and ratio, the main plume would need to be moved higher to match TROPOMI AOCH and the concentration of CO from 1 to 3 km would need to be increased. This would result in a double plume structure with one elevated and one located near the surface. In this profile, the model plume is inconsistent with the observations in multiple ways. The elevated plume is too low in altitude (implying an underestimated injection height at the source) and the surface plume is too low in concentration (implying that too much CO was injected). When used together, the ALH and CrIS/TROPOMI ratio provide much more information about this profile than either could individually. It should be noted that making the adjustments listed above to this plume would likely also improve surface concentrations and reduce the bias found in the model for this profile (see circle markers at the bottom of the panel). Cases like this where CrIS is the most sensitive close to the surface are the minority (Supplementary Fig. S2b); and thus, in most cases, the surface concentration would likely remain unaffected by adjusting the location of an elevated plume to match both the AOCH and the ratio. Improvement of the bias would require other strategies, such as modifications to plume rise parameterization itself or assimilation of surface observations.

Finally, Fig. 8d shows a more complicated case with a modeled double plume structure. This profile is from Southern California on Sept 10th when there was smoke from a fire burning locally and transported smoke from fires burning elsewhere in the state. The model and observational ALH are only 1 km apart, but the ratios show a difference of 0.45. The two model plumes are above and below the altitudes where CrIS is most sensitive which contributes to the model having a much lower ratio than observed. Joining those plumes into one plume centered near 8 km would drastically improve the ratio and raise the model ALH as well. However, it should be noted that profiles with more complicated plume structure make the analysis more uncertain. TROPOMI AOCH has been found to perform worse when plumes are present at multiple altitudes and the limited information from the CrIS/TROPOMI ratio makes it difficult to pinpoint the actual height of the plume or plumes.

## Discussion

As extreme wildfires increase in frequency, accurate modeling of wildfire smoke events grows more important in order to understand and predict effects on climate, ecosystems, and human health. This study evaluates the performance of two state of the art models (RAP-Chem and MOMO-Chem) against surface CO observations, satellite derived CO total column and aerosol layer height retrievals during the 2020 West Coast wildfire event to evaluate the spatial and vertical distribution of wildfire smoke.

RAP-Chem performs with higher skill than MOMO-Chem resolving the CO total column, aerosol layer height, and CrIS/TROPOMI ratio. RAP-Chem overestimates the CO total column but better captures the spatial coverage of smoke while MOMO-Chem shows low correlation and underestimates both the emissions and spatial extent of smoke. When averaged by day, RAP-Chem overestimates the CO total column during most periods of fire activity across all regions whereas MOMO-Chem tends to underestimate during periods of fire activity and misses the peak in CO during periods of extreme fire activity. RAP-Chem has a R^2^ value of 0.54 and 0.37 against the ALH and CrIS/TROPOMI ratio respectively while MOMO-Chem shows low correlation with either metric.

Despite this, MOMO-Chem outperforms RAP-Chem in resolving the surface concentration of CO, however, this analysis is limited by the number of observation sites and their proximity to fires. While both models underestimate the highest CO concentrations over the West region, MOMO-Chem has a lower bias than RAP-Chem because all the emissions are placed at the surface. Scaling the RAP-Chem surface CO to the observed concentrations only marginally affects the ALH and increases the bias against the observed AOCH, indicating that the assimilation of observed AOCH likely would not significantly improve surface concentrations of CO for this event. Current “operational” regional air quality models don’t include chemical data assimilation but are working towards that functionality. Assimilation of AOCH can be further tested in these systems to determine the overall effect. This analysis reveals the limitations of current plume rise parameterizations and how a model can resolve wildfire emissions and injection height well but underestimate the surface concentrations. This has negative implications for surface air quality forecasts and modeling the effects of high concentrations of surface smoke.

The joint use of satellite derived aerosol layer height products and the CrIS/TROPOMI CO ratio can provide information about the height of the plume and relative concentrations at certain altitudes. In the case studies shown, model verification analysis utilizing both metrics can improve the model derived vertical distribution of smoke. Due to the complicated nature of many plume structures, there is a need for a more algorithmic approach to estimating a plume that satisfies both ALH and the CrIS/TROPOMI ratio. This has the potential to also improve surface concentrations in select cases; however, widespread improvement of surface concentrations will require improvements to the plume rise parameterization itself, top-down emissions estimates or assimilation of surface observations. These methods for evaluation of smoke vertical distribution can also be applied to models that explicitly resolve the fire heat flux and convective plume rise.

Wildfires are becoming more frequent and intense which increases the motivation to improve plume rise parameterization to show consistency with observations of plume height, and surface concentrations. This study has focused on vertical smoke distribution in the free troposphere, but stratospheric injection of smoke is also an important component of smoke vertical distribution. Future studies are needed to use measurements of smoke particles in the lower stratosphere to evaluate model performance^49^. The fraction of emissions that are placed at the injection height vs those that remain at the surface could be another area of improvement. RAP-Chem treats this as a fixed value for all fires (10% at the surface, 90% injected) but more variability may be needed. This fraction has been estimated based on limited data available from field campaigns^40^ and may not be indicative of all wildfire behavior. Currently a comprehensive set of measurements to constrain all physical quantities is lacking though there may be some methods for improvement. For example, a satellite derived fire modified combustion efficiency^50^ or a model derived wildfire index such as the Hourly Wildfire Potential (HWP)^51^ could be used to modify this fraction as a function of combustion phase (i.e., smoldering vs. flaming). Regional meteorology and topography may also influence the fraction of smoke injected. Additional sources of observational data including weather radar^52^ may be used to improve the plume injection height and fraction of emissions injected. Another strategy involves trying to fully resolve smoke injection by adding a wildfire heat flux^53,54^. The verification techniques utilized in this study also provide a valuable resource for model verification of the vertical distribution of smoke.

## Methods

### Observation data

TROPOMI, aboard the Copernicus Sentinel-5 Precursor satellite, is a spaceborne nadir-viewing imaging spectrometer that measures backscattered and reflected sunlight from the Earth with a push-broom configuration^55^. It covers wavelength bands between the shortwave infrared (SWIR) and the ultraviolet (UV) with a swath 2600 km wide. The horizontal resolution is typically 7 × 7 km^2^ for SWIR bands though this is dependent on the orbital position and spectral interval. The Sentinel-5P satellite is on a sun-synchronous orbit, crossing the Equator at 13:30. These characteristics give TROPOMI daily global coverage at high resolutions. TROPOMI’s observation of reflected SWIR solar radiation yields atmospheric carbon monoxide measurements that have high sensitivity at all levels of the atmosphere, including the planetary boundary layer^56^. TROPOMI passes over the Western United States in the early afternoon which is just before the typical time for the most intense fire behavior^45,46^. TROPOMI has been validated against aircraft-based vertical profiles^57,58^, satellite^58^, and ground based measurements^59^ and shown good agreement with biases ranging from 1 to 3.73%. When validated during the 2020 Western Wildfire event, TROPOMI had normalized mean biases typically below 24% demonstrating the ability of this retrieval to capture the majority of the CO column^60^. We have used the reprocessed TROPOMI Level 2 data for September 2020.

CrIS, aboard the Suomi NPP satellite^61^, is a Fourier transform spectrometer operating in three spectral bands from 650 to 1095 cm^−1^, 1210 to 1750 cm^−1^, and 2155 to 2550 cm^−1^. The diameter of the ground pixels is 14 km at nadir. The Suomi NPP satellite is also on a near-polar, sun synchronous orbit, crossing the Equator in a similar orbit and only a few minutes after Sentinel-5P. Thus, CrIS also has daily global coverage with retrievals at nearly the same time as TROPOMI. CrIS CO profiles are retrieved using optimal estimation^62,63^ as part of the TRopospheric Ozone and Its Precursors from Earth System Sounding (TROPESS; https://tes.jpl.nasa.gov/tropess) and have been validated extensively against independent data^64^ with biases substantially less than 1%. Diagnostic information provided by TROPESS was used to construct observation operators for comparison with the models^65,66^. These data have been used to understand fires similar to the Western US fires^67^.

TROPOMI Aerosol Optical Central Height (AOCH) is a product developed by Chen et al.^47^ that uses the spectral signature of light absorbed by O_2_ in the A and B bands in TROPOMI retrievals to estimate aerosol layer height over dark targets. AOCH is defined as the peak height assuming aerosol extinction following a quasi-Gaussian distribution and tends to be close to the center height of an aerosol layer. In comparison to the operational TROPOMI ALH product using just the O_2_ A band^68^, this two-band retrieval algorithm shows better performance retrieving the central height of the aerosol layer when compared with spaceborne lidar measurements with a bias of ~0.5 km over land and ocean. We utilized the highest quality operational data, with cloudy pixels filtered out using the slope of the spectral reflectance, the UV aerosol index ≥ 1, and AOD > 0.2.

Surface CO concentrations were obtained from state air quality agency monitoring stations made available through the US EPA Air Quality System. Files containing hourly CO concentrations from 234 stations located across the country were downloaded for September 2020. We only considered measurements taken during the same hour as the satellite overpass to avoid differences due to temporal variability. Data availability posed a challenge as the monitoring stations are not evenly distributed across the country, and there are few stations in the regions most heavily impacted by smoke. This data is considered of the highest quality and has passed several quality control tests.

### Modeling systems

The Rapid Refresh with Chemistry (RAP-Chem) is an experimental air quality forecast model operating at NOAA Global Sciences Laboratory (GSL). It combines the physics and dynamics of the Rapid Refresh Forecast model^69^ with the Weather Research and Forecasting model coupled with Chemistry (WRF-Chem)^70,71^. RAP-Chem uses Fire Radiative Power (FRP) measurements detected within the previous 24 h to estimate wildfire emissions using the same methodology as High Resolution Rapid Refresh with Smoke (HRRR-Smoke)^72^. Emission factors for gasses and aerosols (including CO) are from Andreae (2019)^73^. As in HRRR-Smoke, both the averaged FRP and emissions have a climatological diurnal cycle applied on the biomass burning emissions from the previous 24 h (i.e., persistence). RAP-Chem uses the Freitas plume rise parameterization to simulate smoke injection height, a one-dimensional cloud resolving model that is generally embedded in a 3D host model^74^. Fires are represented as surface buoyancy fluxes that are dependent on fuel type, fire size, and total sensible heat flux. In the model, 10% of emissions are released at the surface with the remaining 90% released into the column as predicted by the plume rise parameterization. Freitas shows better diurnal variation in plume height^75^ and tends to outperform other parameterizations that assign fire emissions to fixed vertical distribution or a single altitude^42,76^. However, Freitas also tends to underestimate the range of plume heights and overpredict injections into the free troposphere^24,26^. Anthropogenic emissions are designed to reflect COVID-19 reductions: the fuel-based oil and gas (FOG) inventory^77^ is used for oil and gas nitrogen oxides (NOx) and volatile organic compounds (VOC) emissions; Areal VCP emissions are modified based on the volatile chemical product (VCP) emission inventory for 2018^78^. Where possible for point sources, we use the Continuous Emission Monitoring System (CEMS) and rely on the National Emissions Inventory 2017 v1 baseline for those sources not reported by CEMS. Outside of the contiguous United States, RAP-Chem uses the Community Emissions Data System 2017 inventory^79^. The emissions have a diurnal profile and are delineated by weekday, Saturday, or Sunday.

The MOMO-Chem framework is a global chemical data assimilation system that integrates multiple satellite observations to constrain atmospheric composition^80,81^. In this study, we used the second version of the Tropospheric Chemistry Reanalysis (TCR-2)^80^, which was produced using the MOMO-Chem framework^81^. TCR-2 employs a state-of-the-art data assimilation approach to integrate information obtained from satellite observations of CO, O₃, NO₂, SO₂, and HNO₃. It simultaneously estimates both atmospheric concentrations of trace gases and surface emissions of key precursors, including CO. The TCR-2 products have been extensively validated against independent observations, such as ozonesonde and aircraft measurements, and have demonstrated good performance in reproducing global distributions of major tropospheric trace gases, including CO and ozone^80^. These products have been used to investigate the impacts of changing anthropogenic and natural activities on tropospheric composition^82–84^. For CO emission estimation, TCR-2 assimilates Measurements of Pollution In The Troposphere (MOPITT) CO data, using a priori emissions taken from the Global Fire Emissions Database (GFED) inventory. However, the limited vertical sensitivity of MOPITT near the surface, combined with retrieval uncertainties and spatial sampling constraints, poses challenges in capturing rapid temporal and spatial variations in CO emissions. Note that none of the observations used for model evaluation in this work are assimilated in this version of MOMO-Chem. The spatial resolution of the global model used in TCR-2 (~1.125° × 1.125°) limits its ability to resolve localized fire events and associated plume structures. Moreover, MOMO-Chem does not include a plume rise parameterization and all biomass burning emissions are placed at the surface level, as in many other global chemical transport models. This limits the system’s ability to accurately represent the vertical transport and dynamics of fire plumes, which can influence the simulation of near-source concentrations and long-range smoke transport.

### CO column calculation

In order to compare the satellite CO total column retrievals with the model output, a column averaging kernel needs to be applied. The column averaging kernel describes the sensitivity the satellite instrument has to CO at each time, location and pressure level and can be applied to modeled vertical profiles to estimate what the satellite would retrieve under those conditions. Both instruments used in this study retrieve CO in the infrared so there will be a minimal effect on the averaging kernels from a layer of smoke aerosols in the column. We estimated the CO total column from the RAP-Chem and MOMO-Chem data by co-locating the model data to the satellite grid and applying the CrIS and TROPOMI averaging kernels. The TROPOMI averaging kernel is derived from a first-order Tikhonov–Phillips regularization on a logarithmic scale^85,86^ and is applied with the following equation:

1 $${\hat{{\boldsymbol{x}}}}_{m,T}={{\boldsymbol{x}}}_{m}{{\boldsymbol{A}}}_{{TROPOMI}}$$

where ${{\boldsymbol{x}}}_{m}$ is a vector containing the regridded and vertically interpolated model CO profile in molecules/m^3^, ${{\boldsymbol{A}}}_{{TROPOMI}}$ is the unitless TROPOMI averaging kernel matrix, and ${\hat{{\boldsymbol{x}}}}_{m,T}$ is the model CO profile transformed by the TROPOMI averaging kernel. The total column in molecules/m^2^ is calculated by multiplying ${\hat{{\boldsymbol{x}}}}_{m,T}$ by the TROPOMI vertical grid spacing (1000 m) and summing over all vertical levels.

The CrIS averaging kernel is derived from Optimal Estimation as the logarithm of the volume mixing ratio that takes the vertical sensitivity of the instrument into account and results in a smooth profile^64^:

2 $${\hat{{\boldsymbol{x}}}}_{m,C}={{\boldsymbol{x}}}_{a}+{{\boldsymbol{A}}}_{{CrIS}}\left({{\boldsymbol{x}}}_{m}-{{\boldsymbol{x}}}_{a}\right)$$

where ${{\boldsymbol{x}}}_{a}$ is the vector containing the a priori profile used in the TROPESS retrieval as the logarithm of the volume mixing ratio, ${{\boldsymbol{x}}}_{m}$ is a vector containing the regridded and vertically interpolated model CO profile as the logarithm of the volume mixing ratio, ${{\boldsymbol{A}}}_{{CrIS}}$ is the CrIS averaging kernel matrix, and ${\hat{{\boldsymbol{x}}}}_{m,C}$ is the model CO profile vector transformed by the CrIS averaging kernel as the logarithm of the volume mixing ratio. The model data was regridded to the satellite grids using the xESMF Python library’s^87^ conservative normalized regridding method.

The resulting model datasets with each satellite averaging kernel applied were converted to consistent units of CO columns (molecules/cm^2^) and all datasets were regridded to a consistent resolution of 1.125 degrees matching the MOMO-Chem grid. These calculations allowed for a direct comparison with the satellite data by creating the same metric from a “virtual retrieval”, i.e., what CrIS or TROPOMI would provide if the world followed RAP-Chem or MOMO-Chem. Surface CO from the model was collocated to the location of surface CO stations using the closest grid cell for direct comparison.

### Aerosol layer height calculation

A model equivalent ALH value was calculated to compare the satellite ALH retrieval with model output. As the TROPOMI AOCH retrieval gives the approximate middle of the aerosol layer, the model equivalent ALH values were calculated by taking a vertical weighted average of aerosols:

3 $${{\boldsymbol{ALH}}}_{m}=\mathop{\sum }\limits_{i=1}^{n}{{\boldsymbol{a}}}_{i}{{\boldsymbol{h}}}_{i}/\mathop{\sum }\limits_{i=1}^{n}{{\boldsymbol{a}}}_{i}$$

where ${{\boldsymbol{ALH}}}_{m}$ is the model equivalent ALH, ${{\boldsymbol{h}}}_{i}$ is the middle height of each grid cell, and ${{\boldsymbol{a}}}_{i}$ is the aerosol profile. Due to data availability, the weighted average was calculated using the extinction coefficient at 550 nm for RAP-Chem (assuming volume averaging in the Mie calculations^88^) and PM_2.5_ concentration for MOMO-Chem. Finally, all datasets were regridded to a consistent resolution of 1.125 degrees.

### Ratio of CO columns

**Fig. 9:**
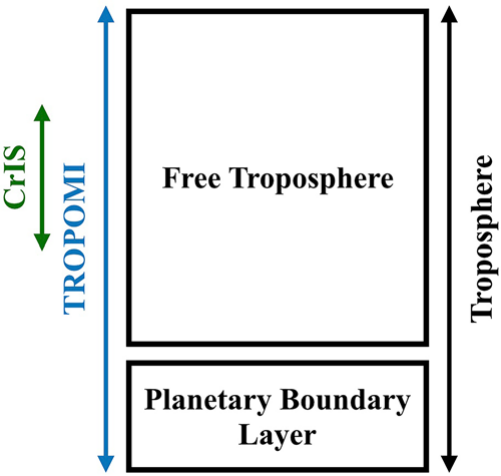
Sensitivity of TROPOMI and CrIS to the troposphere. Schematic of the general sensitivity of TROPOMI and CrIS to the troposphere. TROPOMI has sensitivity to the entire tropospheric column while CrIS is sensitive to a variable range of altitudes in the free troposphere. Taking the ratio between the total CO columns from these instruments give the fraction of the total CO column in the altitudes where CrIS is sensitive.

A new metric was developed to gain more information about the vertical distribution of CO (Fig. 9). We took the ratio between the CrIS CO total column retrieval (***TC***_*C*_) and the TROPOMI CO total column retrieval (***TC***_*T*_) both in units of molecules/cm^2^.

4 $${\boldsymbol{r}}=\frac{{{\boldsymbol{TC}}}_{C}}{{{\boldsymbol{TC}}}_{T}}$$

The CrIS/TROPOMI ratio (***r***) gives the approximate fraction of CO within the altitudes that CrIS is most sensitive to, which was highlighted in previous work as a potentially useful synergistic product between these two sensors^60^.The closely timed overpasses of CrIS and TROPOMI make the retrievals comparable, but their differences in sensitivity is what makes the comparison valuable. This metric provides additional information about the height and vertical distribution of CO. An equivalent calculation was performed for the RAP-Chem and MOMO-Chem outputs with the respective averaging kernels applied for model comparison.

### CrIS vertical sensitivity

We developed a metric to assess the vertical range where CrIS was more sensitive. The CrIS relative sensitivity to different altitudes was calculated by creating a profile with an artificial CO plume centered over three consecutive vertical levels of the model and negligible concentrations otherwise:

5 $${{\boldsymbol{x}}}_{{\boldsymbol{p}},{\boldsymbol{i}}}=\left\{\begin{array}{l}0.01\,ppm\,\,\,\,\,\,\,\,\,\,\,\,\,\,\,\,\,\,\,\,\,\,\,\,\,\,\,\,\,1\le h\le i-1\,\\ 8\,ppm\,\,\,\,\,\,\,\,\,\,\,\,\,\,\,\,\,\,\,\,\,\,\,\,\,\,\,\,\,i-1\le h\le i+1\\ 0.01\,ppm\,\,\,\,\,\,\,\,\,\,\,\,\,\,\,\,\,\,\,\,\,\,\,\,\,i+1\le h\le {n}_{{CrIS}}\end{array}\right.for\,2\le i\le {n}_{{CrIS}}-1$$

where ***x***_***p,i***_ is the artificial profile and ${n}_{{CrIS}}$ is the number of vertical pressure levels ranging from 1211 hPa to 0.1 hPa. The artificial CO plume was over two consecutive levels for the topmost and bottommost levels. The CrIS averaging kernel was applied to this artificial profile using Eq. (2). CrIS’s relative sensitivity to each altitude (***s***_*i*_) was calculated by taking the ratio of the CO total column of the artificial profile after the averaging kernel was applied (***TC***_*A,i*_, see Eq. (2)) and the CO total column of the artificial profile before the averaging kernel was applied (***TC***_*i*_):

6 $${{\boldsymbol{s}}}_{i}=\frac{{{\boldsymbol{TC}}}_{A,i}}{{{\boldsymbol{TC}}}_{i}}{for}1\le i\le {n}_{{CrIS}}$$

This method was repeated by shifting the artificial plume to the other vertical levels to create profiles of the CrIS relative sensitivity to different altitudes at each location referred to as the CrIS vertical sensitivity (***s***). We will use this metric as a tool to interpret the CrIS/TROPOMI ratio. The altitudes where this profile peaks are the altitudes where CrIS has the highest sensitivity; and thus, the CrIS/TROPOMI ratio will provide the fraction of the column in the region where the CrIS vertical sensitivity peaks.

## Supplementary information

Supplementary information

## Data Availability

The surface CO observations are available from the US EPA Air Quality System at https://www.epa.gov/outdoor-air-quality-data. TROPESS CO CrIS at the Goddard Earth Sciences Data and Information Services Center at https://disc.gsfc.nasa.gov/datasets/TRPSYL2COCRSRS_1/summary. TROPOMI CO data was obtained from the Copernicus Data Space Ecosystem at https://dataspace.copernicus.eu/. The Tropospheric Chemical Reanalysis (TCR-2) data are available at https://tes.jpl.nasa.gov/tes/chemical-reanalysis/. The TROPOMI AOCH data is available upon request from Xi Chen(xi-chen-4@uiowa.edu) and Jun Wang(jun-wang-1@uiowa.edu). The RAP-Chem output is available upon request from Jordan Schnell(jordan.schnell@noaa.gov).

## Code availability

Custom code for this manuscript was written to generate all figures presented. Code was written in Python and is available upon request from Mackenzie Arnold (mmarnold7@g.ucla.edu).
