# Supplementary material for: Constraints on the modeled vertical distribution of smoke during the 2020 western US wildfires from satellite data
Source: NPJ Clean Air. 2025 Dec 4;1(1):37. doi: 10.1038/s44407-025-00036-3 (PMC12678182; doi:10.1038/s44407-025-00036-3)
Supplement: Supplementary file 1 — Supplementary information [file 44407_2025_36_MOESM1_ESM.pdf]

Supporting Information for

Constraints on the modeled vertical distribution of smoke during the 2020 western US wildfires from satellite data

Mackenzie M. Arnold[1*], Pablo E. Saide[1,2*], Kazuyuki Mizayaki[3], Kevin W. Bowman[3], Jordan Schnell[4,5], Ravan Ahmadov[5], Xi Chen[6], Jun Wang[6], and Oscar A. Neyra-Nazarrett[2,3]

1. Department of Atmospheric and Oceanic Sciences, University of California, Los Angeles, 2. Institute of the Environment and Sustainability, University of California-Los Angeles, 3. Jet Propulsion Laboratory, California Institute of Technology, Pasadena, CA, USA, 4. Cooperative Institute for Research in Environmental Sciences (CIRES), University of Colorado Boulder, 5. NOAA Global Systems Laboratory, Boulder, Colorado
6. Department of Chemical & Biochemical Engineering, The University of Iowa, Iowa City, IA 52242, USA.

*Corresponding authors: Mackenzie Arnold (mmarnold7@g.ucla.edu), Pablo Saide (saide@atmos.ucla.edu)

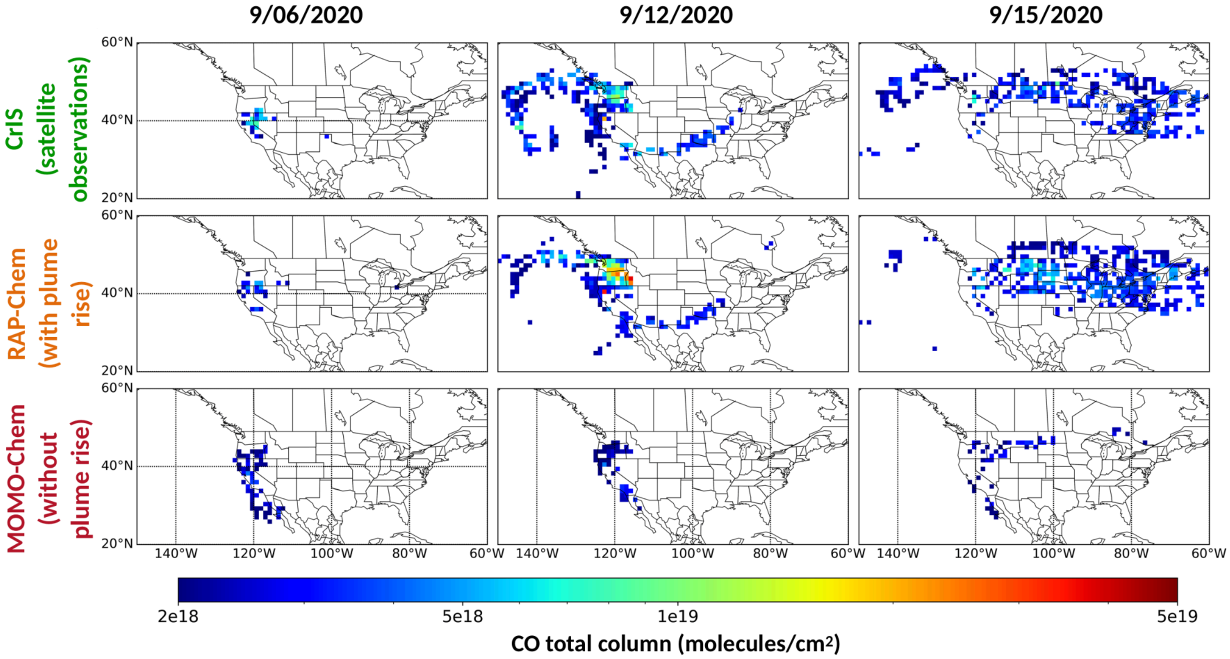

Supplementary Fig. 1 CrIS CO total column, RAP-Chem CO total column with CrIS averaging kernel applied, and MOMO-Chem CO total column with CrIS averaging kernel applied on September 6, 12, and 15, 2020. Units are molecules/cm2. Data is masked by smoke using a TROPOMI CO total column threshold of 3e18 molecules/cm2.

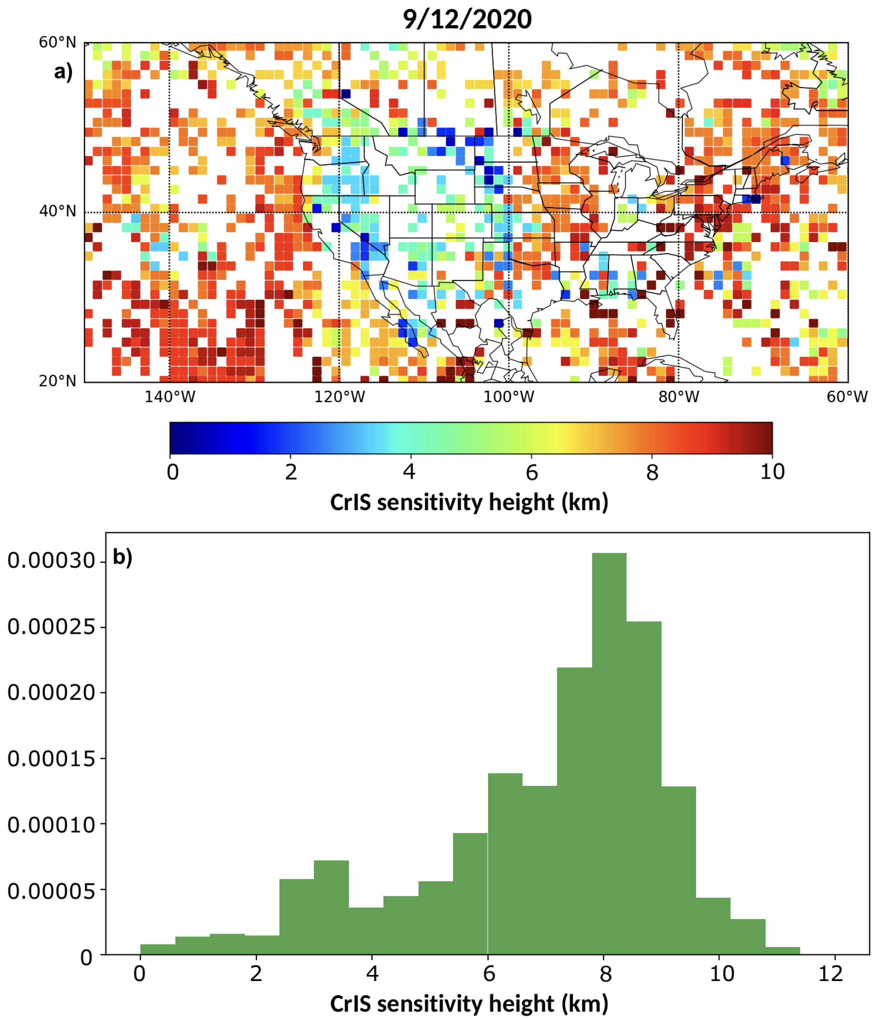

Supplementary Fig. 2 a) Altitude of CrIS maximum sensitivity in meters on September 12, 2020. b) Histogram of the altitude of CrIS maximum sensitivity in meters from September 1-19 2020.

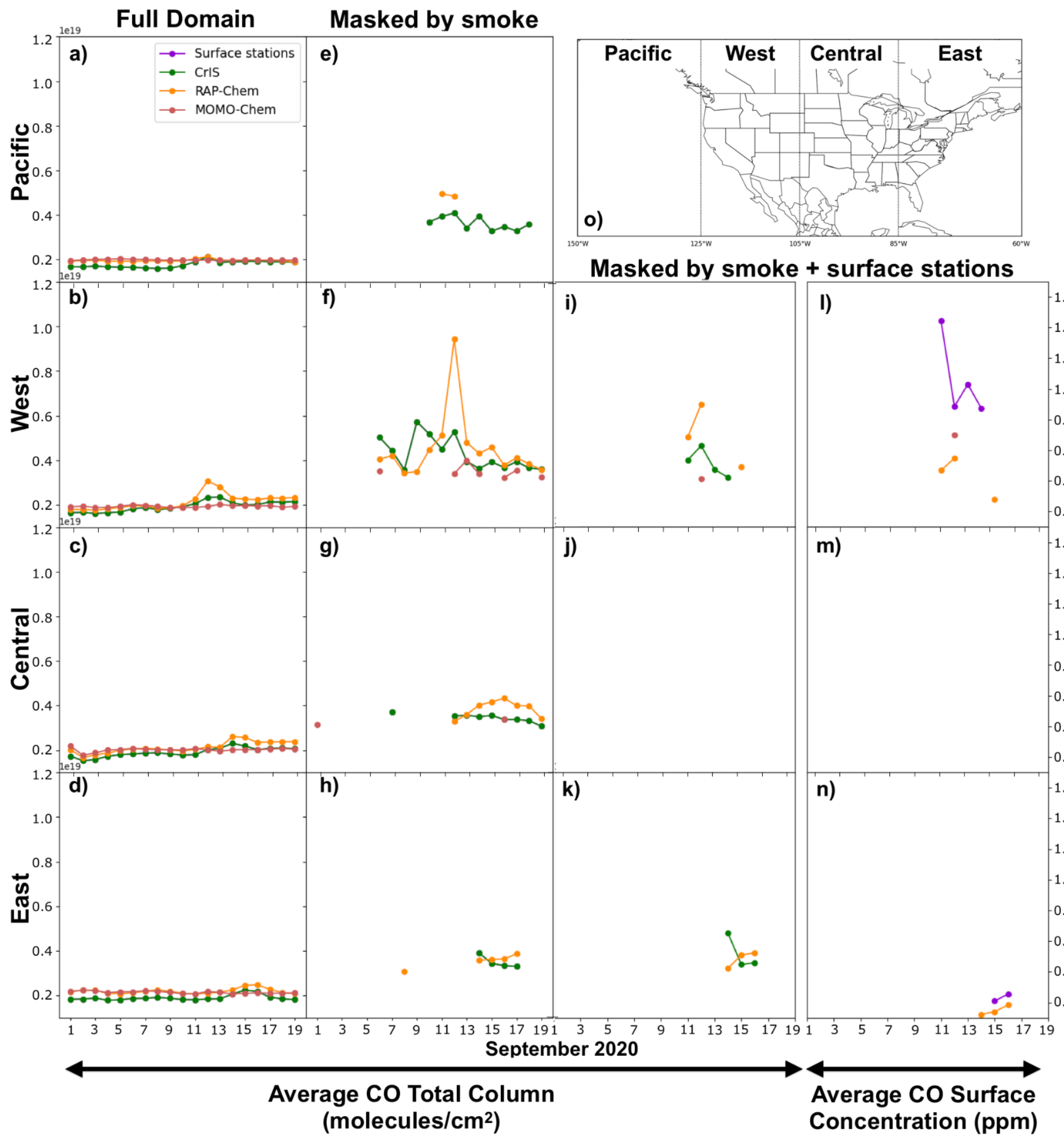

Supplementary Fig. 3 Daily average CO total column in molecules/cm[2] for CrIS, RAP-Chem and MOMO-Chem averaged over the full regional domain (a-d), masked by smoke (e-h), and masked by smoke and surface CO stations (i-k). Daily average CO Surface Concentration for surface CO stations, RAP-Chem and MOMO-Chem (l-n). Model CO total columns have the CrIS averaging kernel applied. Smoke mask is defined by a CO total column threshold of 3e18 molecules/cm[2]. Regions are defined as followed and shown in o): Pacific (150W-125W), West (125W-105W), Central (105W-85W), East (85W-60W).
